# Management of severe immune-related adverse events and outcomes in patients with advanced non-small cell lung cancer receiving immune checkpoint inhibitors

**DOI:** 10.1093/oncolo/oyae318

**Published:** 2024-11-20

**Authors:** Jarushka Naidoo, Douglas B Johnson, Charlotte Doran, Yuexi Wang, Yan Zhang, Trong Kim Le, Sari Hopson, Brian Dreyfus, Lincy S Lal, Charmy Vyas, Shay Goldstein, Zara Izadi

**Affiliations:** RCSI University of Medicine and Health Sciences, Beaumont RCSI Cancer Centre, Dublin 9, Ireland; Beaumont Hospital Dublin, Beaumont RCSI Cancer Centre, Dublin 9, Ireland; Sidney Kimmel Comprehensive Cancer Centre, Johns Hopkins University, Baltimore, MD 21287, United States; Vanderbilt University Medical Center, Nashville, TN 37232, United States; ConcertAI, LLC, Cambridge, MA 02138, United States; ConcertAI, LLC, Cambridge, MA 02138, United States; Bristol Myers Squibb, Princeton, NJ 08540, United States; Bristol Myers Squibb, Princeton, NJ 08540, United States; Bristol Myers Squibb, Princeton, NJ 08540, United States; Bristol Myers Squibb, Princeton, NJ 08540, United States; ConcertAI, LLC, Cambridge, MA 02138, United States; Bristol Myers Squibb, Princeton, NJ 08540, United States; Bristol Myers Squibb, Princeton, NJ 08540, United States; Bristol Myers Squibb, Princeton, NJ 08540, United States

**Keywords:** immune checkpoint inhibitors, non-small cell lung cancer, immune-related adverse events, adverse events management, real-world outcomes

## Abstract

**Background:**

Immune checkpoint inhibitors (ICIs) are associated with severe immune-related adverse events (s-irAEs) that result in hospitalization, emergency department (ED) visits, treatment discontinuation, or death. This study examined the impact of s-irAEs and their earliest management strategies on clinical outcomes in advanced non-small cell lung cancer (NSCLC).

**Methods:**

Data were derived from ConcertAI Patient360 NSCLC, a US-based electronic medical record database, between January 2012 and May 2021. Eligible patients had advanced NSCLC and received ICI-containing therapy. s-irAEs and management actions were abstracted from unstructured EHR data from ICI initiation through the earliest of 100 days after ICI discontinuation, start of a non-ICI-containing regimen, loss to follow up, end of study period, or death. Multivariable Cox regression analysis was used to evaluate the association between s-irAEs and their earliest management strategies, and real-world progression-free survival (rwPFS) and real-world overall survival (rwOS).

**Results:**

The study included 3211 patients. Median (IQR) age was 67 (60-73) years, and 44.9% were female. Most patients (61.6%) initiated ICIs as first-line therapy; half (50.1%) initiated ICIs as monotherapy, with nivolumab monotherapy (29.5%) as the most common initial ICI-containing regimen in any line. Overall, 8.6% of patients experienced s-irAEs, most often diarrhea (3.5%), pneumonitis (1.4%), and rash (1.3%). Among patients who experienced at least one s-irAEs, over half (57.4%) were hospitalized, and 71.8% were treated with corticosteroids, any time after the occurrence of their first s-irAEs. Median rwPFS was 4.9 (95%CI, 4.6-5.2) months, and median rwOS was 13.6 (12.6-14.7) months from ICI initiation. rwPFS and rwOS were comparable between patients with s-irAEs vs patients without s-irAEs when s-irAEs were first managed with anti-cancer treatment interruptions. Patients with s-irAEs had a 53% (22.3%-91.4%) higher risk of death than patients without s-irAEs when s-irAEs initially required corticosteroids or other immunosuppressants, and a 61% (37.9%-87.9%) higher risk of death when s-irAEs first required hospitalization or ED admission.

**Conclusion:**

The impact of s-irAEs on clinical outcomes may depend on the initial intervention required to manage the adverse event. s-irAEs were associated with worse outcomes when they initially required hospital/ED admission, corticosteroids, or other immunosuppression.

**Implications for practice**

This study suggests that among patients with advanced non-small cell lung cancer receiving immune checkpoint inhibitor (ICI) therapy, the initial management of severe immune-related adverse events (s-irAEs) could be an important factor in modifying the impact of s-irAEs on treatment outcomes. Early detection and intervention of s-irAEs in the outpatient setting have the potential to improve patient outcomes by reducing the need for hospitalization or immunosuppression. Further research is needed to validate these findings and to explore the optimal management of irAEs, based on the natural history of AE, patterns of AE, and steroid response.

## Introduction

Immune checkpoint inhibitors (ICIs) that target programmed cell death protein 1 (PD-1) or its ligand (PD-L1) are a class of cancer immunotherapy that are widely used to treat a range of cancers, including advanced non-small cell lung cancer (aNSCLC).^1^ Treatment with ICI therapy for PD-L1-high (>50%) NSCLC improves clinical outcomes compared to chemotherapy.^2^ However, because ICI agents modify the activity of immune cells through the PD-1/PD-L1 pathway, they are associated with immune-related adverse events (irAEs) that can affect multiple organ systems. irAEs are common, occurring in 54%-76% of patients receiving ICI therapy, and range from mild/moderate to severe or potentially life-threatening and fatal.^3^ The most commonly affected organ systems are gastrointestinal and skin, although irAEs can include hepatic, endocrine, pulmonary, renal, central nervous system, cardiac, and other inflammatory events.^4^

Current guidelines for the treatment of irAEs depend on the organ system and severity and include a range of interventions, such as close monitoring of patients, interruption or permanent discontinuation of ICI treatment as well as immunosuppression through administration of corticosteroids (CS) or other immunosuppressive drugs (ISD).^5^ A number of studies have evaluated the association between irAEs and the efficacy of ICIs and found that irAEs may be associated with a better response to therapy.^6,7^ However, this association may be more reflective of low-grade irAEs than severe irAEs (s-irAEs)—as indicated by the provider and/or resulting in hospitalization, outpatient emergency department (ED) visit, treatment discontinuation, or death, as some data suggest s-irAEs are associated with worse clinical outcomes compared with low-grade irAEs.^8^ While mild/moderate irAEs are more common, s-irAEs can substantially disrupt cancer care and have long-term consequences that impact quality of life and clinical outcomes; therefore, early detection and appropriate management are critical.^9^ Research examining the impact of irAE management on ICI clinical outcomes has been limited, especially for s-irAEs, and some evidence suggests that the use of CS at the start or during ICI-containing therapy may negatively impact overall survival (OS) and progression-free survival (PFS).^10-12^

Although prior real-world studies have evaluated patterns of irAE management in community and academic settings,^13,14^ identifying optimal evidence-based strategies for the management of s-irAEs remains a key unmet need. The aim of this study was to evaluate the real-world incidence and management of s-irAEs that occur during ICI-containing therapy and to assess the impact of s-irAEs and their earliest management strategy on clinical outcomes, including real-world PFS (rwPFS) and real-world OS (rwOS).

## Methods

### Patient population

This study used data from the ConcertAI Patient360 NSCLC dataset with additional curation of s-irAEs and management actions using a manual chart review. At the time of the study, the Patient360 NSCLC dataset consisted of structured and unstructured data elements from electronic medical records (EMR) of over 19 000 patients with a confirmed NSCLC diagnosis. The dataset sources clinical data from multiple partners regardless of EMR provider and principally includes community oncology practices (more than 70%) with geographic representation from across the US (with an overall average of ~15% Northeast, ~25% Midwest, ~40% South, and ~20% West). All secondary data were deidentified and involved no direct patient contact, therefore, patient consent was not required. This research project was reviewed by an institutional review board (IRB; Advarra) and found to be exempt from IRB oversight.

**Figure 1.**
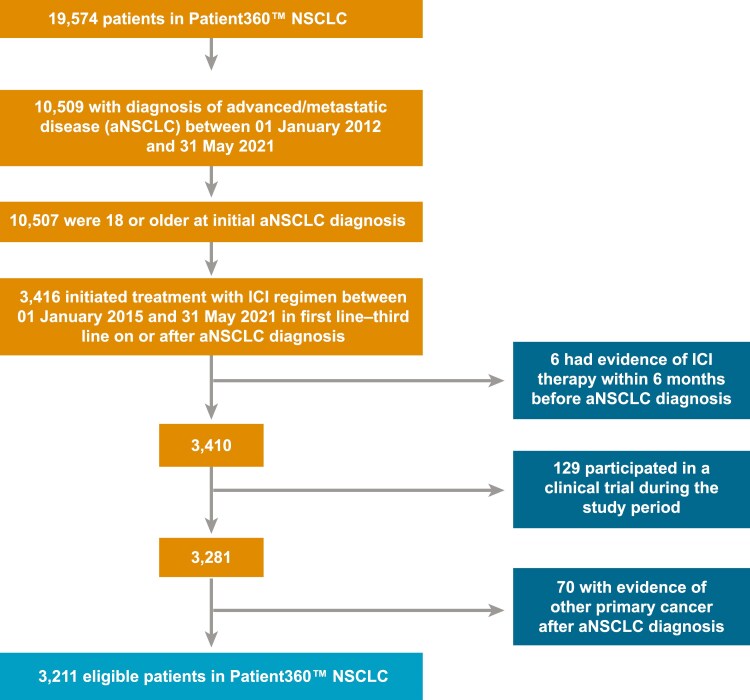
Study attrition. Abbreviation: ICI, immune checkpoint inhibitor; NSCLC, non-small cell lung cancer.

The study included patients with aNSCLC (stage IIIB-IV based on physician documentation; staging edition was not specified in the data) diagnosed between 01 January 2012 and 31 May 2021. For inclusion, patients were additionally required to initiate an ICI-containing regimen that included pembrolizumab, nivolumab, ipilimumab, and/or atezolizumab, alone or in combination with another ICI, chemotherapy, or other systemic anti-cancer agents, in the first 3 lines of therapy after advanced cancer diagnosis, starting from 01 January 2015 (All eligibility criteria in **Figure 1**). A line of therapy was defined as multiple treatments initiated within the same 30-day period, with no agent being discontinued or held for more than 120 days.

### Study design and data collection

**Figure 2.**
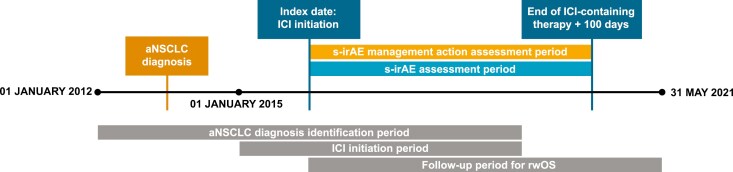
Study timelines. Abbreviation: aNSCLC, advanced non-small cell lung cancer; ICI, immune checkpoint inhibitor; rwOS, real-world overall survival; s-irAE, severe immune-related adverse event.

This study was a retrospective, observational study of patients with aNSCLC initiating ICI-containing therapy. The primary clinical outcomes of interest were rwPFS and rwOS from ICI initiation (index date). For the outcome of rwPFS, patients were followed from index date to the earliest of disease progression or death (due to any cause), end of the medical record, or end of the study period, if the medical record was ongoing (Figure 2). For the outcome of rwOS, patients were followed from index date to the earliest of death (due to any cause), end of the medical record, or end of the study period.

Disease progression events were collected based on provider documentation of progression in unstructured EHR documents. Date of death was confirmed based on several sources, including (in order of source document confirmation), provider documentation, structured EHR data, and third-party death data.

s-irAEs were collected from the index date and were defined as any adverse event (AE) that was associated with hospitalization, outpatient ED visit, early anti-cancer agent/radiation therapy discontinuation, or death, or indicated by the provider as grade 3+, “serious,” or “severe.” Collection of s-irAEs included AEs commonly associated with ICI-containing therapy in the following organ systems: skin, gastrointestinal, endocrine, hepatic, pulmonary, musculoskeletal, renal, and other of interest (full list in Appendix A). Management actions were collected from the date of the first s-irAE and were categorized as treatment discontinuation, treatment hold, administration of CS, administration of ISD other than CS (full list of CS and ISD in Appendix B), and other/undocumented action. s-irAEs and management actions were abstracted using a manual chart review of unstructured physician documentation and reconciled with structured data were appropriate, through to the earliest of 100 days after the end of ICI-containing therapy, start of a non-ICI-containing regimen, end of record, or the end of the study period.

### Statistical analysis

Descriptive statistics were used to describe patient characteristics, incidence of s-irAEs, and management actions. Chi-squared or Fisher’s Exact test was used to compare distributions for categorical variables, and Student’s *t*-test or Kruskal-Wallis test was used for continuous variables. Kaplan-Meier curves were used to analyze time-to-event outcomes.

A multivariable Cox regression analysis was used to evaluate the association between s-irAEs and their earliest management strategy, and outcomes. In patients who had multiple s-irAEs, only the first s-irAE was included in the Cox regression analysis. s-irAEs were modeled as a time-varying covariate and categorized into mutually exclusive groups based on their earliest management action: no s-irAE (reference category), s-irAE and treatment hold/discontinuation, s-irAE and administration of CS/ISD, s-irAE and hospitalization or outpatient ED visit, and s-irAE and other/unknown management strategy. Other covariates which included baseline demographics and clinical characteristics were selected using bidirectional stepwise regression with a threshold of *α* = 0.10. Age, race, and gender were included in the Cox model regardless of statistical significance. Results were considered statistically significant at *α* = 0.05.

## Results

### Patient characteristics

A total of 3211 patients were included, and the latest date of follow-up in the cohort was 13 May 2021. The median (IQR) age of patients at ICI initiation was 67 (60-73) years, 44.9% were female, and 79.2% were White. The median (IQR) duration of follow-up from ICI initiation to the end of the study period was 12.3 (3.3-17.5) months. Most patients (81.2%) had advanced disease at initial NSCLC diagnosis, and the most common sites of distant metastasis were bone (34.4%), brain (19.7%), and lung (16.9%). Over one-third of patients (35.9%) had chronic obstructive pulmonary disease at advanced diagnosis, and 15.5% had diabetes (Table 1). About half of the patients (50.1%) initiated ICIs as monotherapy, with nivolumab monotherapy (29.5%) as the most common initial ICI regimen in any line, followed by pembrolizumab +  carboplatin + pemetrexed (29.2%). Most patients (61.6%) initiated ICI-containing therapy in 1L, with pembrolizumab + carboplatin + pemetrexed (42.6%) as the most common regimen in 1L, followed by pembrolizumab monotherapy (22.8%).

**Table 1.** Baseline characteristics of patients with advanced NSCLC initiating ICI-containing therapy.

Variable (*N* = 3211)	Statistic
Age at ICI initiation, years	
Mean (SD)	66.5 (9.7)
Median (IQR)	67 (60,73)
Female gender, *n* (%)	1442 (44.9)
Race, *n* (%)	
Black or African American	420 (13.1)
White	2544 (79.2)
Other	247 (7.7)
Ethnicity, *n* (%)	
Hispanic or Latino	46 (1.4)
Not Hispanic or Latino	2812 (87.6)
Missing/undocumented	353 (11.0)
Follow-up from ICI initiation, months	
Mean (SD)	12.4 (12.5)
Median (IQR)	12.3 (3.3, 17.5)
Year of ICI initiation, *n* (%)	
2015	179 (5.6)
2016	335 (10.4)
2017	493 (15.4)
2018	624 (19.4)
2019	816 (25.4)
2020	660 (20.6)
2021	104 (3.2)
*Initial diagnosis*	
Stage, *n* (%)	
Stage I-IIIA or III (NOS)	420 (13.1)
Stage IIIB-IV	2632 (81.2)
Missing/undocumented	159 (5.0)
Disease histology, *n* (%)	
Adenocarcinomas	1818 (56.6)
Squamous cell neoplasms	680 (21.2)
Other	565 (17.6)
Missing/undocumented	148 (4.6)
*Advanced diagnosis*	
Stage, *n* (%)	
All stage III	296 (9.2)
Stage IIIB	270 (8.4)
Stage IIIC	26 (0.8)
All stage IV	2755 (85.8)
Missing/undocumented	160 (5.0)
Sites of distant metastasis, *n* (%)	
Bone	1103 (34.4)
Brain	634 (19.7)
Lung	544 (16.9)
Liver	466 (14.5)
Adrenal gland	373 (11.6)
ECOG performance status, *n* (%)	
<2	2287 (71.2)
≥2	420 (13.1)
Missing/undocumented	504 (15.7)
Comorbid conditions at advanced diagnosis, *n* (%)	
Chronic obstructive pulmonary disease	1152 (35.9)
Diabetes	496 (15.5)
Renal disease	120 (3.7)
*Index date*	
Body mass index^a^, *n* (%)	
Underweight (<20)	420 (13.1)
Normal (20-24.9)	1084 (33.8)
Overweight (25-29.9)	914 (28.5)
Obese (≥30)	650 (20.2)
Missing/undocumented	143 (4.5)
Lactate dehydrogenase (LDH) results^b^, *n* (%)	
Abnormal	703 (21.9)
Normal	374 (11.7)
Missing/undocumented	2134 (66.5)
Aspartate aminotransferase (AST) results^c^, *n* (%)	
Abnormal	264 (8.2)
Normal	2883 (89.8)
Missing/undocumented	64 (2.0)
Alanine aminotransferase (ALT) results^d^, *n* (%)	
Abnormal	229 (7.1)
Normal	2781 (86.6)
Missing/undocumented	201 (6.3)

^a^Body mass index is extracted directly from the medical record and calculated based on height and weight information.

^b^Normal range for LDH was defined as 100-190 U/L.

^c^Normal range for AST was defined as 8-48 U/L for males and 8-43 U/L for females.

^d^Normal range for ALT was defined as 7-55 U/L for males and 7-45 U/L for females.

Abbreviations: ECOG, Eastern Cooperative Oncology Group; ICI, immune checkpoint inhibitor; IQR, interquartile range; NOS, not otherwise specified; NSCLC, non-small cell lung cancer; SD, standard deviation.

### Incidence of severe immune-related adverse events and management strategy

Overall, 277 patients (8.6%) experienced at least one s-irAE during ICI therapy, most often gastrointestinal (3.9%) or skin (1.8%). s-irAEs that occurred most frequently included diarrhea (3.5%), pneumonitis (1.4%), and rash (1.3%) (**Table 2**). The median (IQR) time from treatment initiation to the first s-irAE was 2.8 (1.1-7.0) months overall, 3.6 (1.4-8.8) months in patients initiating ICI monotherapy, and 2.3 (0.9-5.4) months in patients initiating ICI combination therapy. Among patients who experienced at least one s-irAE, over half (57.4%) were hospitalized, a quarter (24.6%) were seen in an outpatient ED, 71.8% were treated with CS, 2.5% were treated with other ISDs, 20.6% had an anti-cancer agent held, and 2.2% had an anti-cancer agent discontinued, any time after the occurrence of their first s-irAE (Table 3). Most patients received a management action on the same day as their first s-irAE (78.0%).

**Table 2.** Incidence of s-irAEs among patients with advanced NSCLC initiating ICI-containing therapy.

Variable (*N* = 3211)	Statistic
Any s-irAE^a,b^, *n* (%)	277 (8.6)
Time to first s-irAE, months	
Median (IQR)	2.8 (1.1, 7.0)
Skin, *n* (%)	58 (1.8)
Rash	42 (1.3)
Pruritus	18 (0.6)
Gastrointestinal, *n* (%)	124 (3.9)
Diarrhea	111 (3.5)
Colitis	16 (0.5)
Endocrine, *n* (%)	14 (0.4)
Hypothyroidism	8 (0.3)
Adrenal insufficiency	6 (0.2)
Hepatic, *n* (%)	7 (0.2)
Hepatitis	4 (0.1)
Hepatotoxicity	1 (0.0)
Liver enzyme increase	2 (0.1)
Pulmonary, *n* (%)	51 (1.6)
Pneumonitis	46 (1.4)
Interstitial lung disease	5 (0.2)
Musculoskeletal, *n* (%)	5 (0.2)
Inflammatory arthritis	5 (0.2)
Renal, *n* (%)	24 (0.8)
Nephritis	1 (0.0)
Renal failure	3 (0.1)
Acute kidney injury	21 (0.7)
Other s-irAEs of interest, *n* (%)	12 (0.4)
Cardiac conditions^c^	9 (0.3)
Pancreatitis	3 (0.1)

^a^All occurrences of s-irAEs were evaluated from the index date through to the earliest of 100 days after the discontinuation of ICI therapy, initiation of non-ICI therapy, end of record or death, or end of the study period.

^b^The following adverse events were examined in this study but were not identified in the data: vitiligo, hyperthyroidism, new onset diabetes, hypophysitis, drug-induced liver injury, encephalitis, uveitis, Guillain-Barre syndrome, and myasthenic syndrome.

^c^Cardiac conditions included myocarditis, pericarditis, and vasculitis. For pericarditis and vasculitis, only s-irAEs associated with sentinel events were included in the analysis. Sentinel events were defined as hospitalization, emergency department visit, early anti-cancer agent discontinuation, early radiotherapy discontinuation, or death.

Abbreviations: ICI, immune checkpoint inhibitor; IQR, Interquartile range; NSCLC , non-small cell lung cancer; s-irAE, severe immune-related adverse event.

**Table 3.** All management actions any time^a^ after the first s-irAE among advanced NSCLC patients initiating ICI-containing therapy who experienced at least one s-irAE.

Variable (*N* = 277)	Statistic
Management action, number of patients (%)	
Hospitalization	159 (57.4)
Emergency department visit	68 (24.6)
Anti-cancer agent hold	57 (20.6)
Anti-cancer agent discontinuation	6 (2.2)
Administration of corticosteroids	199 (71.8)
Administration of other immunosuppressive drugs	7 (2.5)
Other or unknown management action	13 (4.7)

^a^Management actions were collected from the date of the first s-irAE through to the earliest of 100 days after the end of ICI-containing therapy, start of a non-ICI-containing regimen, end of record or death, or the end of the study period.

Abbreviations: ICI, immune checkpoint inhibitor; NSCLC, non-small cell lung cancer; s-irAE, severe immune-related adverse event.

### Clinical outcomes

**Figure 3.**
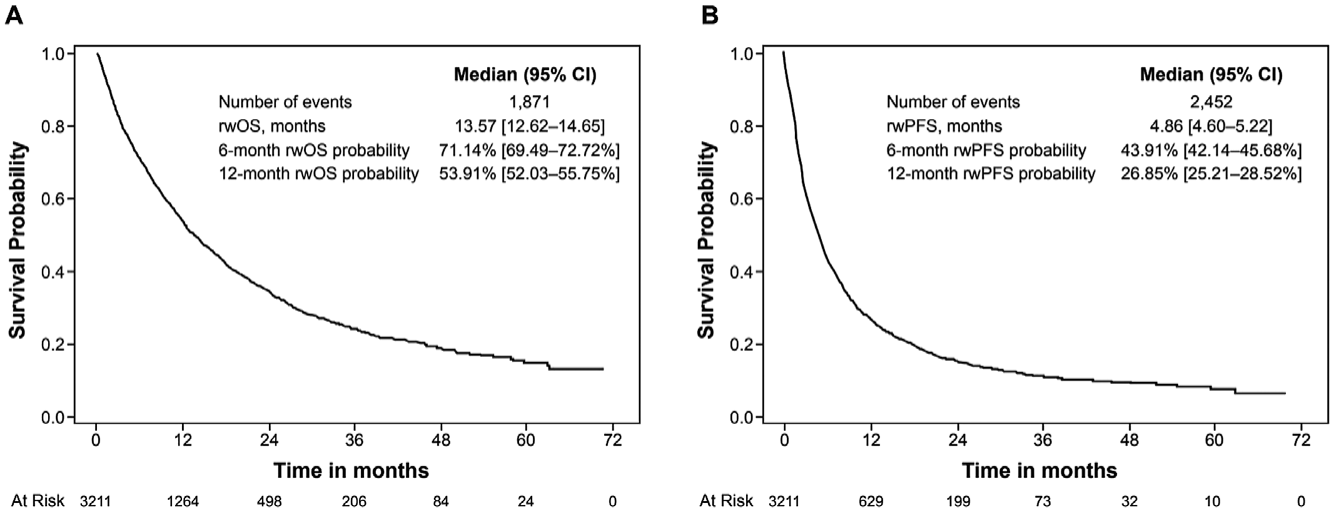
Kaplan-Meier analysis of (A) rwOS and (B) rwPFS from the start of ICI-containing therapy among patients with advanced NSCLC. CI, confidence interval; ICI, immune checkpoint inhibitor; NSCLC,  non-small cell lung cancer; rwOS, real-world overall survival; rwPFS, real-world progression-free survival.

The unadjusted median rwPFS was 4.9 (95% CI, 4.6-5.2) months from ICI initiation and by 6 months, patients had a 43.9% chance of remaining progression-free (Figure 3). In adjusted analyses, patients with s-irAEs first managed with CS/ISD showed a 37.4% increased risk of progression or death (95% CI, 11.7%-69.0%) compared to patients who did not experience s-irAEs. Patients with s-irAEs first managed with anti-cancer treatment hold or discontinuation had a similar risk of progression or death compared to patients who did not experience s-irAEs (Adjusted hazard ratio [HR_adj_] 0.812, 95% CI, 0.598-1.102). Patients who experienced s-irAEs that first required management in a hospital or outpatient ED setting had a 28.9% increased rate of progression or death (95% CI, 11.7%-48.8%) compared to patients without s-irAEs (Table 4).

**Table 4.** Multivariable Cox regression analysis of rwPFS and rwOS from ICI initiation among patients with advanced NSCLC.

		rwPFS			rwOS		
Variables^a^	Stratum N	Hazard ratio	95% Confidence interval	*P*-value	Hazard ratio	95% Confidence interval	*P*-value
s-irAE and earliest management action (ref: no s-irAE)							
s-irAE with anti-cancer agent discontinuation or treatment hold	38	0.812	0.598-1.102	.1820	0.955	0.697-1.310	.777
s-irAE with CS/ISD	71	1.374	1.117-1.690	.0027	1.530	1.223-1.914	.0002
s-irAE first managed in hospital or outpatient ED setting	155	1.289	1.117-1.488	.0005	1.610	1.379-1.879	<.0001
s-irAE with other or unknown management action	13	1.060	0.662-1.698	.8069	1.284	0.783-2.107	.3218
Age < 65 years at index date (ref: age ≥65 years)	1,366	0.921	0.851-0.998	.0436	0.810	0.740-0.886	<.0001
Male gender (ref: female gender)	1,769	1.096	1.012-1.186	.0233	1.118	1.022-1.223	.0145
White race (ref: non-white)	2,544	1.047	0.949-1.155	.3567	1.112	0.991-1.246	.0704
ICI therapy regimen (ref: nivolumab)							
Pembrolizumab monotherapy	598	0.736	0.643-0.842	<.0001	—	—	—
Atezolizumab monotherapy	63	1.091	0.831-1.431	.5319	—	—	—
ICI + ICI	138	1.065	0.865-1.310	.5541	—	—	—
Other ICI combination	1,448	0.805	0.709-0.915	.0008	-	—	—
2L+initiation of ICI therapy (ref: 1L)	1,232	1.149	1.036-1.274	.0083	1.203	1.100-1.316	<.0001
Histology at initial diagnosis (ref: adenoma/adenocarcinoma)							
Squamous cell carcinoma	680	1.119	1.013-1.236	.0274	1.269	1.136-1.418	<.0001
Other or unknown	713	1.076	0.975-1.188	.1470	1.129	1.011-1.260	.0306
BMI (kg/m^2^) closest to index date (ref: BMI≥25)							
BMI <25	1504	1.104	1.020-1.196	.0146	—	—	—
Unknown	143	1.101	0.903-1.342	.3422	—	—	—
Year of index date on or after 2018 (ref: before 2018)	2204	0.913	0.826-1.008	.0722	—	—	—
Sites of metastasis							
Bone (ref: no evidence of bone metastases)	1103	1.209	1.114-1.311	<.0001	1.176	1.073-1.290	.0005
Liver (ref: no evidence of liver metastases)	466	1.469	1.318-1.637	<.0001	1.432	1.272-1.612	<.0001
Performance status (ref: ECOG ≥ 2)							
ECOG < 2	2,287	0.820	0.729-0.922	.0009	.702	0.617-0.798	<.0001
Unknown	504	0.789	0.681-0.913	.0015	.648	0.549-0.764	<.0001
							
Comorbid conditions							
COPD (ref: no COPD)	1152	-	-	—	1.142	1.042-1.251	.0045
Lab tests							
Normal LDH (ref: LDH not in normal range)	374	0.830	0.720-0.955	.0094	.754	0.643-0.884	.0005
Unknown (ref: LDH not in normal range)	2,134	0.892	0.810-0.981	.0186	.878	0.790-0.975	.0155
Normal AST (ref: AST not in normal range)	2883	0.757	0.657-0.871	.0001	.687	0.591-0.799	<.0001
Unknown (ref: AST not in normal range)	64	0.823	0.602-1.127	.2250	.686	0.487-0.967	.0313

^a^Baseline demographic and clinical characteristics were considered as potential confounders if they were statistically significant at *α* = 0.10 in univariate analyses. Bidirectional stepwise selection was used to select covariates for inclusion in the final model with a threshold of *α* = 0.10, and results were considered statistically significant at *α* = 0.05. Age, race, and gender were included in the models regardless of statistical significance.

Abbreviations: 1L/2L, first line/second line; AST, aspartate aminotransferase; BMI, body mass index; COPD, chronic obstructive pulmonary disease; CS, corticosteroids; ECOG, Eastern Cooperative Oncology Group; ED, emergency department; ICI, immune checkpoint inhibitor; ISD, immunosuppressive drugs; LDH, lactate dehydrogenase; NSCLC, non-small cell lung cancer; rwOS, real-world overall survival; rwPFS, real-world progression-free survival; s-irAE, severe immune-related adverse event.

A total of 1871 patients died during the study period, and unadjusted median rwOS was 13.6 (95% CI, 12.6-14.7) months. By 6 months from ICI initiation, the survival probability was 71.1% (95% CI, 69.5%-72.7%) (Figure 3). Similar to findings for rwPFS, patients with s-irAEs first managed with CS/ISD showed a 53.0% increased risk of death from any cause (95% CI, 22.3%-91.4%) compared to patients who did not experience s-irAEs after adjusting for baseline characteristics. Patients with s-irAEs first managed with anti-cancer treatment hold or discontinuation had a similar risk of death to patients who did not experience s-irAEs (HR_adj_ 0.955, 95% CI, 0.697-1.310). Patients who experienced s-irAEs that first required management via hospitalization or outpatient ED visit had a 61.0% increased risk of death (95% CI, 37.9%-87.9%) compared to patients with no s-irAEs (**Table 4**).

## Discussion

To the best of our knowledge, this is the largest real-world study to examine the association between s-irAEs and their management, and clinical outcomes in patients with aNSCLC. This study found that 8.6% of patients with aNSCLC treated with ICI-containing therapy in the real-world clinical setting experienced s-irAEs. Most patients who experienced s-irAEs had them managed with CS/ISD and contrary to guideline-recommendations,^5^ we found that only 23% of patients experiencing at least one s-irAE had a documented interruption to their anti-cancer therapy. The results showed that patients with s-irAEs had worse outcomes than patients without s-irAEs if s-irAEs were first treated with CS/ISD or required initial management in a hospital/outpatient ED setting. However, rwPFS and rwOS were comparable between patients with s-irAEs and patients without s-irAEs if s-irAEs were first managed with cancer treatment interruptions. These findings provide valuable insight into s-irAEs associated with ICI therapy, their management, and clinical outcomes in real-world community oncology practices.

irAEs of any grade have been reported in over half of patients on ICI therapy in prior real-world studies.^13,14^ In a recent study, Guezour et al investigated grades 3-4 irAEs in 201 patients with aNSCLC treated with ICIs from 2 health centers in France and reported these events in 17.9% of patients.^7^ Consistent with our findings, the most commonly affected organ systems were skin, gastrointestinal, and pulmonary.^7^ Overall, s-irAEs developed in a smaller proportion of patients in our study, however, gastrointestinal events were more common than those reported by Guezour et al, which may be due to the fact that we included diarrhea—the most common s-irAE observed in our dataset—as a gastrointestinal event in addition to colitis.

Consistent with published real-world literature we found that administration of CS was the most common s-irAE management strategy^5,14^ and proportions of patients with irAE-related ICI treatment holds previously reported are comparable with our findings.^14^ We observed a smaller proportion of patients with s-irAE-related discontinuations of anti-cancer therapy, compared with ICI discontinuations reported in studies by Teimouri et al^14^ and Brown et al^13^ which explored irAE management strategies in smaller datasets. Discordance in discontinuation rates may be explained by differences in study design and definitions of follow-up as well as differences in the determination of permanent discontinuations (eg, a manual chart review of physician-documented discontinuations vs the use of structured medication fields from claims data). Only 2.2% of patients had their s-irAE managed with permanent discontinuation of anti-cancer therapy in our study. Therefore, the impact of treatment interruptions on study outcomes more accurately reflects the impact of temporary treatment rather than permanent discontinuations of anti-cancer therapy.

A number of studies, including prospective and retrospective research and secondary analyses of clinical trial data, have suggested that patients who experience irAEs have overall favorable treatment outcomes.^15-24^ However, these findings must be interpreted with caution due to methodological challenges and limited generalizability. For instance, some studies have used Kaplan-Meier curves stratified by AE occurrence at any time during the follow-up.^15,18,19,21-23^ This approach can induce immortal time bias, as patients must survive to experience irAEs, leading to a spurious association between irAEs and better outcomes.^25^ A possible solution is to use modified Kaplan-Meier curves that account for time-varying exposures. A multivariable Cox proportional hazard model with irAEs as time-varying covariates is a more robust approach that can also accommodate time-varying confounders in non-randomized settings. Das et al conducted a multicenter retrospective cohort study of 76 patients with gastrointestinal cancers who had received anti‐PD‐1 therapies for US Food and Drug Administration‐approved indications.^15^ While irAEs appeared to be associated with improved outcomes based on results from standard Cox models, the association was not observed in time-dependent covariate analyses.^15^

Another consideration is irAE severity, an important confounder that can impact treatment outcomes. Some prior studies have not accounted for irAE severity,^17^ while others have predominantly included grade 1/2 irAEs, with limited generalizability to higher grades.^16,19,20^ Eggermont et al conducted a secondary analysis of data from a clinical trial of pembrolizumab in advanced melanoma.^16^ The occurrence of irAEs was associated with a longer recurrence-free survival in the pembrolizumab arm, however, this association was not observed in a subgroup analysis of grades 3/4 irAEs.^16^ Similarly, von Pawel et al evaluated treatment outcomes in patients with and without irAEs using data from a randomized trial of atezolizumab in NSCLC.^24^ Authors concluded that OS was in favor of patients with irAEs vs those without irAEs; however, irAEs were mostly grade 1/2, and most patients did not receive CS therapy. Consistent with our findings, CS administration was associated with shorter OS in this study.^24^ To the best of our knowledge our study is the first to systematically investigate how differences in early irAE management modify the relationship between irAEs and treatment outcomes.

We found that over half of patients experiencing at least 1 s-irAE, were hospitalized at some point after their first s-irAE. We also found that patients who experienced s-irAEs that first required management via hospitalization or outpatient ED admission had a 61% higher risk of mortality than patients who did not experience s-irAEs. This suggests that close monitoring and early multidisciplinary intervention in the outpatient setting could potentially assist in timely diagnosis, faster resolution of symptoms, and improved outcomes, particularly for patients at high risk of irAEs (eg, those on combination immunotherapy), and potentially also be cost saving if inpatient hospitalization can be avoided. To balance the benefits of immunotherapy with potential risks, patient education on toxicities, shared decision making, and the significance of early reporting are becoming increasingly crucial.^26^ Effective management of irAEs calls for a multidisciplinary approach, and communication with other medical disciplines is critical for prompt intervention.^27^

One limitation of this study is the potential for unmeasured confounding by specific s-irAE types, more granular grading of s-irAEs, or contraindications for various management strategies. s-irAEs evaluated in this study resemble the profile of irAEs associated with ICI treatment. However, due to the unavailability of treating physician ascertainment, it is possible that s-irAEs reported in this study, although contemporaneous with ICI treatment, were not considered to be immune-related. s-irAEs treated only with treatment hold might represent overestimation of grade by the treating physician and/or not have been irAEs at all. Additionally, the list of irAEs used in this study may not capture all the possible irAEs that patients may experience during immunotherapy. A temporal association was used to relate the first s-irAEs with their corresponding first management actions. It is possible that the actual and the documented date of s-irAEs and/or management actions differed, or the observed management action was not causally related to the occurrence of the first s-irAE. Additionally, delayed irAEs occurring more than 100 days after the discontinuation of ICI treatment were not included in this study. However, these potential misclassifications most likely occurred in a non-differential manner with respect to the outcome, and as such, would bias the results towards the null. Finally, the data used in this study were obtained primarily in the community oncology setting, which may not fully represent the wider underlying population of patients.

## Conclusion

This study demonstrates that the initial management of s-irAEs influences their subsequent impact on treatment outcomes and emphasizes the need for enhanced strategies to optimize patient outcomes through prompt and adequate intervention. We found that patients with s-irAEs had worse outcomes than patients without s-irAEs if s-irAEs required initial management in a hospital/outpatient ED setting or were first treated with CS/ISD. However, outcomes were comparable between patients with and patients without s-irAEs if s-irAEs were first managed with anti-cancer treatment interruptions.

## Data Availability

ConcertAI does not make datasets publicly available because study data are used under license from source practices and other data providers. ConcertAI will consider requests to access study datasets on a case-by-case basis. Please contact us with any inquiries at https://www.concertai.com/contact-us/.

## Appendix A. List of severe immune-related adverse events.

**Table:**

Organ system	Severe immune-related adverse events
Skin	• Rash
	• Pruritis
	• Vitiligo
Gastrointestinal	• Diarrhea
	• Colitis
Endocrine	• New or worsening hypothyroidism
	• Hyperthyroidism
	• Hypophysitis
	• Adrenal insufficiency
	• New onset insulin-dependent diabetes
Hepatic	• Hepatitis
	• Hepatotoxicity
	• Liver enzyme increase (based on provider documentation)
	• Drug-induced liver injury
Pulmonary	• Pneumonitis
	• Interstitial lung disease
Musculoskeletal	• Inflammatory arthritis
Renal	• Nephritis
	• Renal failure
	• Acute kidney injury
Other	• Cardiac conditions
	• Encephalitis
	• Uveitis
	• Pancreatitis
	• Guillain-Barre syndrome
	• Myasthenic syndrome

## Appendix B. List of corticosteroids and immunosuppressive drugs.

**Table:**

Class	Agent
**Corticosteroid (CS)**	Prednisone, Prednisolone, Methylprednisolone, Hydrocortisone, Budesonide, Dexamethasone, Meprednisone, Fludrocortisone, Cortisone, Triamcinolone, Betamethasone
**Immunosuppressive Drugs (ISD)**	Adalimumab, Antilymphocyte Immunoglobulin, Apremilast, Azathioprine, Basiliximab, Cyclophosphamide, Cyclosporine, Everolimus, Fluocortolone, Infliximab, Infliximab Biosimilar 1, Infliximab-Dyyb, Leflunomide, Lenalidomide, Lymphocyte Immune Globulin, Methotrexate, Methotrexate Sodium, Mycophenolate Mofetil, Mycophenolic Acid, Pirfenidone, Pomalidomide, Rituximab, Sirolimus, Tacrolimus, Temsirolimus, Thalidomide, Tocilizumab, Tofacitinib, Ustekinumab, Immune globulin intravenous, Antithymocyte Globulin
